# Computational discrimination between natural images based on gaze during mental imagery

**DOI:** 10.1038/s41598-020-69807-0

**Published:** 2020-08-03

**Authors:** Xi Wang, Andreas Ley, Sebastian Koch, James Hays, Kenneth Holmqvist, Marc Alexa

**Affiliations:** 1 Faculty IV - Electrical Engineering and Computer Science, TU Berlin, Berlin, 10587 Germany; 2 School of Interactive Computing, College of Computing, Georgia Institute of Technology, Atlanta, GA 30332 USA; 3 Department of Psychology, Universität Regensburg, 93053 Regensburg, Germany; 4 Department of Psychology, Nicolaus Copernicus University, 87-100 Toruń, Poland

**Keywords:** Human behaviour, Computer science

## Abstract

When retrieving image from memory, humans usually move their eyes spontaneously as if the image were in front of them. Such eye movements correlate strongly with the spatial layout of the recalled image content and function as memory cues facilitating the retrieval procedure. However, how close the correlation is between imagery eye movements and the eye movements while looking at the original image is unclear so far. In this work we first quantify the similarity of eye movements between recalling an image and encoding the same image, followed by the investigation on whether comparing such pairs of eye movements can be used for computational image retrieval. Our results show that computational image retrieval based on eye movements during spontaneous imagery is feasible. Furthermore, we show that such a retrieval approach can be generalized to unseen images.

## Introduction

Research on eye movements during visual imagery has a long history. Early investigations^1–3^ from the beginning of the twentieth century have established a link between eye movements during visual imagery and mental images, based on observations of eye movements during imagery. Neisser^4^ argued that eye movements are actively associated with the construction of a visual image, and Hebb^5^ suggested that eye movements during imagery and memory retrieval are necessary to assemble and organize “part images” into a whole visualized image.

Many recent studies^6–14^ have shown that humans spontaneously move their eyes when recalling a scene from memory and that such eye movement patterns closely resemble the spatial arrangement of the elements of the recalled content. This effect has not only been demonstrated for participants who encode visual scenes and later recall those scenes while looking at an empty screen^6,7,9,11,14,15^, but also for participants who encode verbal information in association with a spatial cue and later recall such information while looking at an empty screen^12,13^. Moreover, it has been shown that participants who listen to scene descriptions also make eye movements which correspond to spatial positions from the described scene^7,14,15^.

Previous studies evaluated the functionality of eye movements to blank space. Results suggest that eye movements while looking at nothing play a functional role during memory retrieval, and act as “spatial indices”^4,5,11,12,16^ that may provide assistance in memorizing the spacial layout of a scene^4–6,11^. However, it has been argued whether such eye movements can facilitate memory retrieval as additional cues^17,18^. In support of such a functional role, impaired episodic memory performance has been reported in experiments where participants were restricted to look at a fixation cross during recall^9–11,15^. In Johansson et al.^9^, participants’ gaze directions were manipulated towards positions on an empty screen that are either overlapped with the original locations of the to-be-retrieved visuospatial information or selected randomly at irrelevant locations. Results demonstrated that the likelihood of successful memorization is increased when there was an encoding-retrieval overlap in gaze locations. Corresponding results were reported in a follow-up study where similar gaze manipulations were used for participants, who recalled verbal information that had previously been encoded in association with a particular space^13^.

Imagery movements have been reported for other muscles beyond the ocular ones, and a consistent finding is that muscle activation during imagery is a fraction of the activation during action-related period^3,19^. Eye movements during imagery are the only muscular activities that largely replicate the activities during actual perception.

The close similarity between eye movements during perception and those during imagery appear to open up the possibility for computational image retrieval: Use imagery eye movements to pick the image where the similarity between perceptual eye movements and imagery eye movements is the largest. In view of the very robust findings on imagery eye movements, this may at first seem like a trivial task, but there are four reported issues that pose challenges for this task.

- Even though eye movements during mental imagery play a functional role, they do *not* reinstate the eye movements made during encoding of the image^8,13^.
- It has been shown that eye movements during imagery might be potentially driven by covert attention, which may explain the functionality of eye movements while looking at nothing^20^.
- It is also known that recalling an image with closed eyes might be preferable for some of us. This effect has not yet been fully understood^21,22^, and has posted a challenge in practice for eye movement recording using video-based eye trackers.
- Many previous studies consistently reported that eye movements while looking at nothing contain a large distortion due to the lack of reference frame. In particular, the area spanned by eye movements during mental imagery is down scaled, comparing to the area spanned by eye movements during encoding while the visual image is in sight^6–8,15,23^. A clue to why some people scale down their images was given by^8,15^, in which all participants were tested for working memory capacity and the OSIVQ^24^ was used as an assessment for individual differences in object imagery, spatial imagery and verbal cognitive style. Scaling down imagery eye movements was most pronounced with participants who had high scores on spatial imagery. Eye movements during visual imagery tasks are employed to reduce cognitive resources associated with the processing of spatial information, and a weaker spatial imagery ability increases the need for those eye movements.

Image retrieval would only be possible if there is a strong similarity between the eye movements on real versus imagined images across different viewers. Previous studies mainly used simple grid-based stimuli^9,11,13^, where eye movements were discretized into unnaturally low resolution. A more high-resolution scene was used in^7,15^, but nevertheless a single stimuli lacks variation. It is unclear whether s strong similarity in eye-movements remains during encoding and recall of a large amount of *nature images*. In view of the fact that such a similarity exists but its strength was not quantified, we decided to test the following cases:

1. *Encoding vs encoding*: Can a visual image be computationally discriminated from 100 other images, based only on the gaze pattern from observers looking at those images?
2. *Imagery vs imagery*: Can a visual image be computationally discriminated from 100 other images, based only on the imagery gaze pattern from observers asked to think about those images?
3. *Generalizability*: Assuming that we have a classifier that can do 1 and 2, will it be able to computationally discriminate a set of new images, that come with new gaze patterns? In other words, will the retrieval technique generalize?

## Results

**Figure 1:**
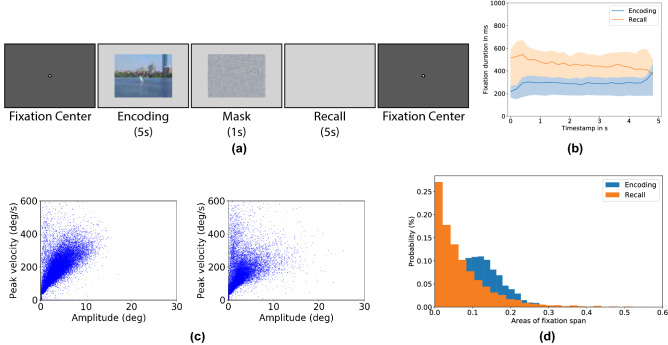
Experimental paradigm and eye movement statistics. (**a**) Paradigm of one single trial in the experiment. After an initial fixation (of 500 ms) at the center of the display, one image stimulus was presented for 5 s, followed by a noise mask. Briefly after that, observers were asked to recall the image they had just seen for another 5 s. (**b**) Average of fixation duration in encoding (blue) and recall (orange). X-axis indicates the time and y-axis corresponds to the duration of each fixation. The black curves in the middle of each plot correspond to the average duration within the five seconds and the light coloured areas indicate the center intervals of 50%. (**c**) Main sequence diagrams during encoding (left) and recalling (right) where peak velocity is plotted as a function of the amplitude of saccades. (**d**) Histogram of the spatial coverage of all fixations over the 100 stimuli. For each image, one bounding box of all fixations in one sequence is drawn and we compute the percentage of the area covered by the bounding box with respect to the whole screen. The distribution of the spatial coverage of all encoding fixations is drawn in blue and the distribution computed from all recall fixations is drawn in orange. (**a** contains public domain imagery downloaded from the dataset published in Judd et al.^45^).

We adopted the encoding-recall experimental paradigm from^9,11^. Observers were instructed to encode a set of 100 images and then to recall them in front of an empty screen. Figure 1a shows an exemplary structure of one trial. Using a video-based eye tracker, we recorded observers’ eye movements during both encoding and recall. Our goal was to quantify the similarity between encoding and recall eye movements in the context of image retrieval. We first evaluated the eye movement parameters and then tested the retrieval performance in the above-mentioned three scenarios, namely encoding vs. encoding, imagery vs. imagery, and generalizability on unseen images. In the end we looked at the results in relation to individual behavior and image content to investigate how they influence the accuracy of the retrieval task.

### Characteristics of eye movements on real and mental images

Three measurements were used to analyze the eye movement sequences: the main sequence graphs of saccades, the fixation count and duration, and the overall spatial coverage. Figure 1c shows the main sequence graphs of saccades during encoding (left) and recall (right), where peak velocity is plotted as a function of amplitude. Imagery saccades are shorter and slower. Fixation duration is plotted over time in Fig. 1b. On average each encoding eye movement sequence has 16 (SD = 2.8) fixations, which have an averaged duration of 278.0 ms (SD = 73.4 ms). Each recall sequence has 11 (SD = 3.6) fixations on average with a mean duration of 452.2 ms (SD = 308.0 ms). Recall sequences have less fixations than encoding sequences ($t(5.6e3) = 62.77, p<0.001$, Welch’s *t*-test) and recall fixations are longer than encoding ones ($t(6.7e4) = -57.29, p<0.001$, Welch’s *t*-test). Similar observations have been made in previous studies^7,9^ that information retrieval from memory might account for the longer duration of fixations made during mental imagery.

Similar to previous findings in^7,15,23^, we also observed a shrinkage of eye movements on mental images, as shown in Fig. 1d. Unlike fixations during encoding, which are perfectly aligned with the corresponding visual content, fixations during recall very often do *not* coincide with the intended elements in the original image. Consequently, such distortion makes it difficult to estimate the intended locations from recall fixations alone.

### Image retrieval based on eye movements during encoding

We modeled the retrieval of an image based on eye movements as a classification task, in which each single image represents one class. 2D gaze density histogram was used to represent the eye movement sequence. The whole space is divided into a regular grid and the number of raw samples is counted in each cell, which reflects the duration one has fixated in one cell. Note that all sequence information was disregarded in this representation, a choice motivated by repeated findings that encoding positions are revisited during recall but that the sequence is not reinstated. Given an input query, the algorithm estimates the image (class) and the accuracy of each query was evaluated by the rank of the true image (class) among all images (classes). Accumulating all ranks together yielded the accumulated classification rank curve, which essentially is a Receiver Operating Characteristic (ROC) curve. A good retrieval algorithm finds the right image among the top ranked returns, and the corresponds ROC curve quickly reaches the highest point already at the beginning. The retrieval accuracy was measured by the standard area under the curve (AUC).

As a baseline, we classified images using a simple k-nearest neighbor (kNN) classifier based on Euclidean distance. The standard leave-one-out cross validation was used to determine the accuracy, and for each fold, one subject was dropped from the training set and used for the testing only. We reported the averaged results over all cross validation sets. For comparison, we employed a convolutional neural network (CNN) following the structure proposed in Ref.^25^.

The retrieval accuracy of both CNN and kNN were significantly above chance (AUC = $50\%$). Similar to previous results^25^, CNN achieved the accuracy of AUC = $97.5\%$ and AUC = $94.5\%$ was achieved when using kNN. The achieved top-1 and top-3 ranks using CNN were $61.3\%$ and $79.1\%$ respectively, and $60.6\%$ and $77.9\%$ using kNN. This suggests that gaze positions from observers looking at images contain ample information for classification, which, for a database of around 100 images, can be used to retrieve the images with decent accuracy. Even a simple classifier like kNN can correctly identify the visual stimulus more than $60\%$ of the time.

**Figure 2:**
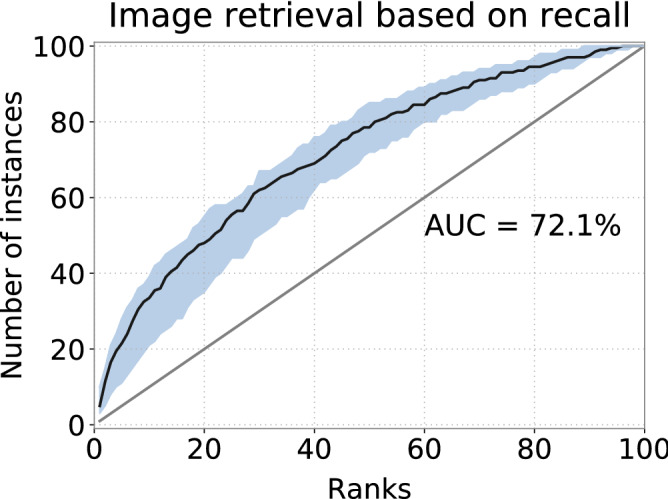
Recall-based retrieval using CNN trained with two tasks. The black curve shows the averaged result of all leave-one-out tests. Light areas depict the $50\%$ intervals of all ROC curves.

**Figure 3:**
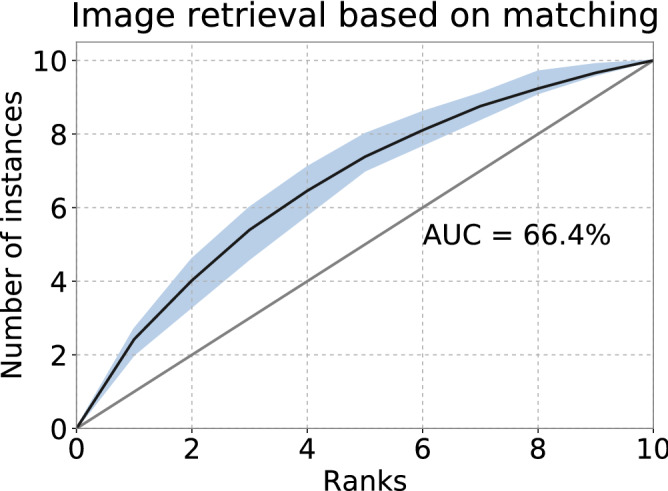
Generalization to unknown stimuli. ROC curve of retrieval based on matched eye movements during recalling. Blue area depicts the 50% intervals of all ROC curves of each subject.

### Image retrieval based on eye movements during recall

We then analyzed the ability of the classifier to retrieve images based on recall eye movements, again using the same leave-one-out cross validation. The performance for the kNN classifier dropped down to chance level (AUC = $54.3\%$, $U=5.5e3$, $n_1=n_2=100$, $p=0.245$ two-tailed, Mann–Whitney rank test). This failure reflects the repeated finding that eye movements during mental imagery suffer from spatial distortion^6,15,26^, so we should not use the Euclidean distance measures on imagery data, unless we could somehow remap the distorted gaze data onto the (unknown) correct positions. Using the same architecture as in encoding, the CNN classifier achieved an accuracy of AUC = $69.8\%$. The top-1 and top-3 rank dropped to $5.9\%$ and $13.8\%$, however, still significantly above the chance performance ($1\%$ and $3\%$).

Even though gaze positions during recall are distorted, they still share many similarities with gaze positions during encoding. To improve the recall-based retrieval, we introduced a second learning task, which forces the network to explicitly learn the mapping of gaze positions between encoding and recall. A decoder was added to the classification network (Fig. 7a). Given a recall gaze histogram, the decoder network was trained to generate an encoding gaze histogram for the same image. Using the same leave-one-out test, we achieved a slightly improved performance of AUC = $72.1\%$ as shown in Fig. 2 ($U=7.3e3$, $n_1=n_2=100$, $p<.001$ two-tailed, Mann–Whitney rank test). We interpret this poor performance to reflect the previously noted variation among observers’ recall behaviour (more discussions in the next section).

### Generalized retrieval of new image content

To investigate the generalizability of the system, we used two networks similar to the ones used for classification to learn 16-dimensional descriptors from the eye movement histograms. Following the architecture proposed in^25^, we used triplet loss^27^ to force descriptors of matching encoding and recall gaze histograms to be closer together than non-matching ones, without learning to map between them. The resulting dual networks allowed us to see whether retrieval based on eye movements during mental imagery can generalize to new image content that is not in the training dataset used by the networks. In other words, whether the system can generalize to unseen images (with eye movement data).

We thus matched recall eye movements to encoding eye movements, and a recall histogram is then assigned to the class of the matching encoding histogram. Matching was restricted to the pairs for each observer and a 10-fold cross validation over the images was used to determine the retrieval accuracy, e.g., for each fold training on 90 images and testing on the remaining 10. An average AUC of $66.4\%$ was achieved over the 10-folds ($U=78.5$, $n_1=n_2=10$, $p=0.250$ two-tailed, Mann–Whitney rank test) (Fig. 3). The top-1 and top-3 ranks were $23.5\%$ and $40.2\%$ respectively. These results indicate that the learned space can be used to describe eye movements related to unseen images, and allows the generalization about new stimuli with a reasonable retrieval accuracy.

### Dependence on individual participants’ behavior

**Figure 4:**
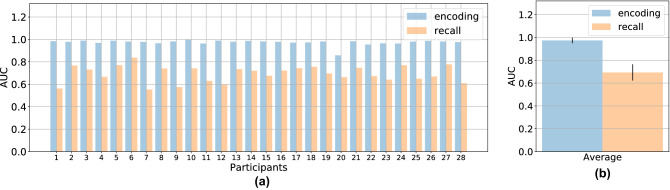
Area under the curve of each leave-one-out test using CNN. (**a**) AUC when data of each subject is used for testing. Classification based on encoding eye movements are shown in blue and classification based on recall eye movements are shown in orange. The mean values and standard errors (one standard deviations) of the AUCs are shown in (**b**).

We examined the variation of the retrieval accuracy among individual dataset and looked at the achieved AUCs from previous leave-one-out cross validation using CNN (Fig. 4a). On average, recall-based retrieval had a larger variation (SD = 0.07) then encoding-based retrieval (SD = 0.02) as depicted in Fig. 4b. It seems that imagery vs imagery retrieval works better for some observers who actively moved their eyes during mental imagery. Our intuition is the current neural network design works well when observers resemble their perceptional eye movements during recall, but performs poorly when it comes to severe distortion, i.e. when eye movements during recall are largely shifted, scaled or translated, or when observers don’t move their eyes much during recall.

### Dependence on stimuli content

Images used in the experiment have 1,024 pixels in the largest dimension and gaze position histograms of eye movements are essentially down-scaled representations. This poses a natural limitation on the total number of images that could be computationally discriminated. As histograms encode information of where we look and how long we look, two distinct images could inevitably lead to two histograms that are very similar to each other. To further investigate this aspect, we visually inspected the most and least misclassified image pairs when CNN was used as the retrieval method.

**Figure 5:**
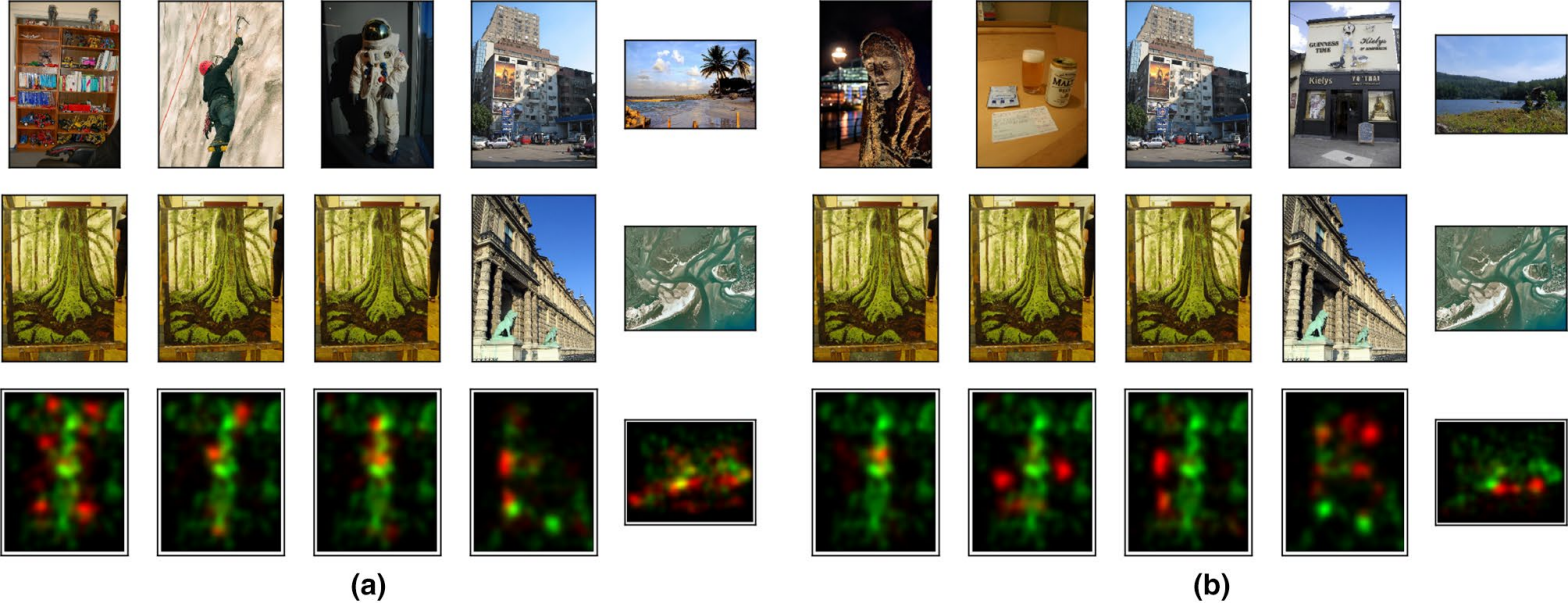
Top-5 image pairs that are most often confused (**a**) and never confused (**b**) in encoding-based retrieval using CNN. (**a**) Image pairs are sorted by the number of confusions from left to right: the first column shows the two images for which the eye movements during encoding are confused most often. In each pair, the image in first row is classified as the image in the second row. Histograms aggregated over the complete dataset are shown in the third row. Histograms of the images in the first row are show in red and histograms of the images in the second row in green. (**b**) Examples of the most distinct pairs of images, i.e., images in the first row are never misclassified as images in the second row. Similar to (**a**), aggregated histograms are plotted in the third row. (Public domain imagery downloaded from the dataset published in Judd et al.^45^).

**Figure 6:**
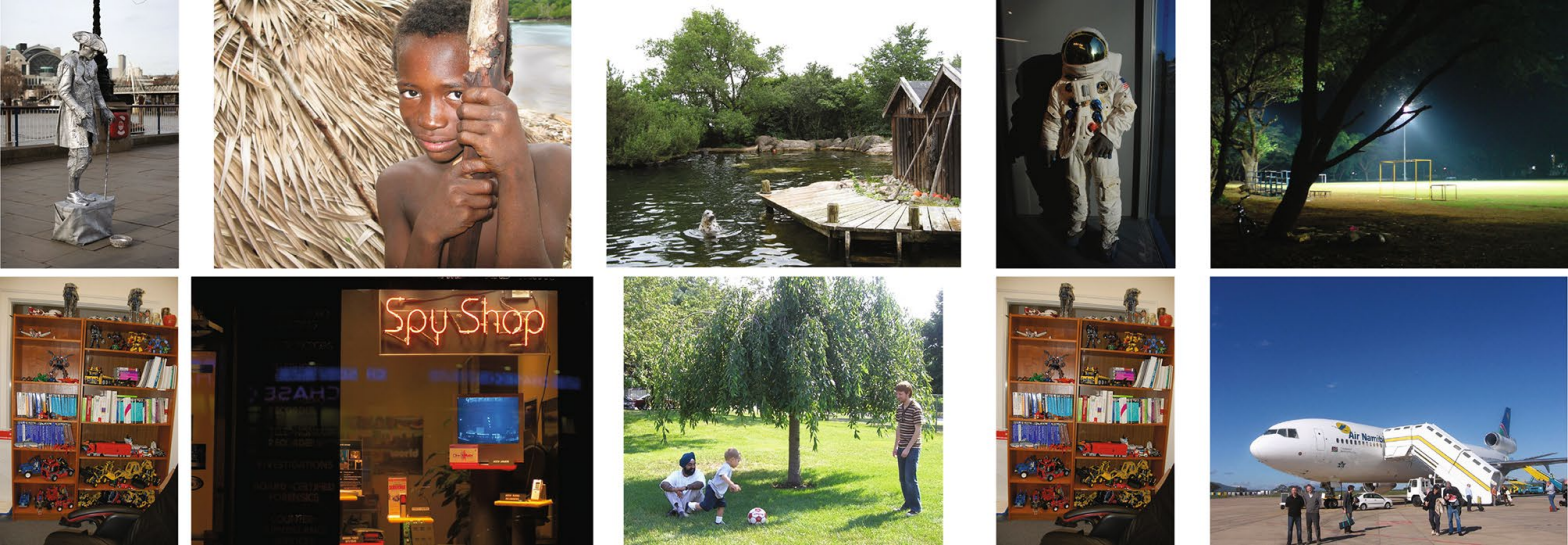
Top-5 most confused image pairs (in each column) in recall-based retrieval. Images in the first row are most often misclassified as the images below. The frequency of each pair’s confusion goes down from left to right. Notably that interesting scene elements in the images of each pair have very similar layout. For example, the two images in the second column have dominant features in the right half: the boy in the top image and the text and display in the bottom one. In the third pair, a dog and a house are placed similarly to the positions where the boy and the adult are in the bottom image. (Public domain imagery downloaded from the dataset published in Judd et al.^45^).

For encoding-based retrieval, the most confusing image pairs (Fig. 5a) had noticeably higher similarities especially in the scene layout, whereas the least confusing pairs (Fig. 5b) shared less similarity. Interestingly, similar results were observed in recall-based retrieval and Fig. 6 shows the most confusing image pairs. Despite the inaccuracy of eye movements during mental imagery, the similarity of image content still seems to be one source of confusion for the classification.

## Discussion

Our study aimed at investigating the possibility of using computational models to retrieve natural images based on eye movements during encoding and mental imagery. We found that image retrieval based on eye movements when looking at the images can achieve very high accuracy even with a simple method. The gaze patterns from observers inspecting the images contain unique signatures of the content, and they can be used to retrieve the corresponding images. However, a successful retrieval based on eye movements when looking at nothing requires more complicated machine learning algorithms. Using a convolutional neural networks (CNN), we demonstrated that eye movements during mental imagery can be used to retrieve images with a reasonable performance. Furthermore, our results show that the intrinsic similarity between eye movements during encoding and those during recall allows us to extend the retrieval framework to new photos.

Using physiological measurements for the retrieval of mental imagery is not new. Others have aimed at reading a person’s mental state^28,29^ or reconstructing the imagery they have in mind^26,30,31^, by making use of brain activities measured by functional magnetic resonance imaging (fMRI). Eye movements during looking at images have also been previously used in practice. In human-computer interaction, fixations were used as a mouse cursor for selection^32^. In Ref.^33^, gaze data were used to indicate where observers allocate their attention in a fine-grained object classification task, as gaze data provided the information on where humans look for the distinction. Surprisingly, eye movements have not been used to retrieve the mental imagery before, despite substantial previous work demonstrating that eye movements during recall in front of an empty space are abundant and play a functional role in memory retrieval^6–14^.

We have replicated previous research showing that gaze patterns from observers encoding images differ from the patterns while observers think about the same images^6–8,15,23^: imagery eye movements cover a smaller area compared to the area covered by encoding eye movements (Fig. 1d), and imagery fixations are longer (Fig. 1b). For this reason, the discriminatory information contained in imagery eye movements is poorer. More specifically, successful discrimination does not work with straight-forward k-nearest-neighbor search (kNN) and its Euclidean distance measure, while convolutional neural networks (CNN) can better discriminate the content. In contrast, gaze patterns from encoding the photographical content contain sufficient information that allow both the kNN and the CNN methods to perform successful discrimination. By explicitly modelling the distortions contained in recall eye movements, the dual-task CNN method achieved an improvement on recall-based retrieval comparing to the pure classification network. Additionally, our analysis results indicate that the retrieval performance varies among individuals and similarities of image content will pose a major challenge of extending to larger databases.

### Eye movements representations

In this study, eye movements were represented with a $24\times 24$ 2D histogram of gaze positions (also known as a gridded gaze density map). This is a natural choice, as most imagery research has shown that the location of fixations is preserved during imagery but not the fixation order. However, other types of information might well be suppressed. Besides the simple 2D histograms, we experimented with several different representations, which are (1) binary histogram excluding the time spent in each cell; (2) histograms with a third dimension of time; (3) concatenated histograms of differences between consecutive eye positions. None of them performed significantly better. It is therefore unclear how much better the image discrimination would be if order information were to be included in the representation of eye movement sequences. We suspect observers may have difficulties to recall anything else than locations of objects in the limited five seconds time. But it is also possible that other details of the mental imagery (e.g. color and texture) are hidden in finer gaze patterns, which might require longer recall time. An interesting direction in future work is to explore different representations for other types of information.

### Improvement on performance

We noticed that image retrieval based on eye movements from recall worked better for some observers than for others. Johansson et al.^8^ showed that people with a poorer spatial imagery ability produced eye movements during imagery with a larger dispersion, more similar to those during encoding of the physical image. People with a good spatial imagery ability can recall photos without making extensive eye movements, which often results in scaled, shifted and translated gaze areas. From this perspective, computational retrieval of image content using eye movements better suits people with a poorer spatial imagery ability.

The down-scaled eye movements during mental imagery make the retrieval task more difficult as clusters are formed in close range around the image center. Maybe we could instruct observers to extensively look around and actively make larger eye movements in the full extent of the tracking space, in other words, to imagine an up-scaled photo. Asking participants to make an effort is not unreasonable and in many brain-computer interface applications, it is the norm. It is known that a successful brain scan using fMRI requires the participants to lie still during imaging. Electroencephalogram (EEG) provides a way to communicate with less physical constrains, especially for patients with aggravating conditions^34,35^. However, the measured signals very often can only be used to distinguish in binary cases^36^. Even though it requires a long training time^37^, blink or other body movements can still lead to low signal-to-noise ratio^38^. In comparison, making eye movements during mental imagery is much more natural and intuitive, and has a potential to be a product that many people could use.

In practice, additional noise could be introduced into the data that lead to a decreased signal-to-noise ratio. For instance, in the uncontrolled experimental condition during mental imagery, participants may easily lose their focus and instead think about something irrelevant to the task. However, such cases are difficult to detect. Without an effective approach to increase the signal-to-noise ratio, both training and testing procedures cannot be free from the impact of noisy data. Due to the lack of reference frame, the retrieval methods suffer from distortions contained in eye movements during mental imagery. Errors in recall gaze locations may accumulate overtime as no feedback was given in the recall period. We did not find any consistent distortion patterns among the recall eye movements even for one dataset of one observer. This could explain why the explicit learning of the mapping between recall and encoding did not significantly improve the performance. More details can be found in the supplementary information 1.

Surprisingly, in the third case (the generalization result), the resulting AUC (Fig. 3) was somewhat weaker than in the second case (the imagery result). We were uncertain about the reason of this performance drop but we suspected this is likely caused by the increased difficulty of having to deal with variations in the encoding *and* imagery data simultaneously. We decided not to include more components than 16 in the descriptor vector, as they would just increase the likelihood to overfit in the network. Eye movements have been used to refer where people allocate their attention for a long time and they are partially driven by the visual features in the images. Studies on visual feature importance^39,40^ could offer a guideline for the integration of image content in the next steps. As the recalled mental imagery potentially shows the encoded scene content in the episodic memory, work on image memorability^41,42^ could be specially relevant in future work.

### Scalability

Photos that have comparable layouts lead to similar gaze patterns from observers looking at them. In the context of computational image retrieval, it means such photos are more likely to be indistinguishable. Our results suggest that such dependencies were also present in the imagery vs. imagery retrieval. For example, a textual sign with a box underneath it results in a similar triangular shape in the gaze data as a photo of face. Clearly, this imposes a natural limit on the number of images a computational method could possibly retrieve based on the eye movements. It does not come as a surprise as eye movements only encode the location and likely the significance of the scene elements. Unless other types of information, e.g. semantics, are available, it remains difficult to differentiate images of similar layout, especially when discrete histograms are used. Yet the computation retrieval method based on eye movements has significantly increased the number of distinguishable image instances. In comparison, brain activities measured by fMRI have only been used to distinguish among smaller datasets with fewer instances. Study in Ref.^43^ used only three film events as testing data and the work^44^ on reconstruction of face images were tested on 30 samples.

Partially due to its high cost of acquisition, fMRI data are often collected from few participants (10 in Ref.^43^ and 6 in Ref.^44^). The sparse dataset may pose a severe challenge when using deep learning methods.

### Long-term memory

The current study only focuses on the effect of short-term memory where image recall was performed immediately after the inspection of the original images. As indicated by previous study^11^, recall/imagery eye movements are likely to be less accurate as memory deteriorates over time, and consequently the retrieval performance would decrease. We have not explored this and it could be an interesting future direction.

## Conclusions

Taken together, our results suggest that it is possible to retrieve an image based on encoding and recall eye movements. Without additional training and practice, naive participants could use our method to retrieve an image. We cannot estimate the feasibility when significantly more images are included in the database, however, the presented results achieved a clear performance benefit over brain activity based measurements. Fewer restrictions are posed on the participants’ side, and if we ask them to exercise a small effort to actively imagine a larger image, the recall-based retrieval is very likely to achieve a comparable accuracy to when encoding eye movements are used.

## Methods

### Participants

Twenty-eight naive participants took part in the study (9 females, mean age 26 ± 4). All participants had normal or corrected to normal vision and none of them had colour deficiencies. They all gave their written informed consent before the experiment and their time was compensated. The experimental protocol was approved by the local Ethics Committee at the Faculty IV of Technische Universität Berlin in compliance with the Guidelines of the German Research Foundation (DFG) on Ethical Conduct for Research involving humans.

### Apparatus

The experiment was conducted in a dark and quiet room. Participants were seated in front of a 24-inch display (resolution: $1{,}920 \times 1{,}200$ pixels; physical size: 0.52 m $\times$ 0.32 m; distance: 0.7 m) on which image stimuli were presented. Each participant viewed and recalled 100 images binocularly, however, only the dominant eye movement was recorded with an EyeLink 1,000 in remote mode at 1,000 Hz. Gaze point on the screen was calibrated using a standard 9-point calibration and a chin and forehead rest was used to help participants stabilize their positions.

### Visual stimuli

We randomly selected 100 images from the MIT data set^45^, including both indoor and outdoor scenes. In practice, an eye-tracking experiment session must have a limited amount of time. Therefore, we decided to use only a subset of 100 images in this study considering our experimental design. All images were presented at the center of display in their original size and the largest dimension has 1,024 pixels.

### Experimental procedure

We followed the standard looking-at-nothing paradigm^9,11^ to collect eye movements during both encoding and recall as shown in Fig. 1a. Observers were instructed to look closely at each image and try to remember its content. The instruction for recall was to think about the image and generate a visual representation of the content which is interesting. Each observer had an initial round of 10 practice trials. We assumed that they had then learned the task, and after that there were no further instructions regarding the task.

The total 100 trials were divided into five blocks and for each participant, images were presented in a randomized order. At the beginning of each block of 20 trials, we calibrated the eye tracker on the display. The calibration procedure was repeated unless it achieved a good accuracy (below $0.5^\circ$) in the following validation. We had 200 eye movement sequences from each participant, including 100 sequences of encoding 100 images and 100 sequences of recall all the images.

### Memory task

At the end of the experiment, we randomly selected five from the viewed 100 images and presented them together with five new unseen images in a randomized order. We asked participants to report whether one image has been seen before. All participants could easily determine which image had been seen before, except for one single mistake made by one participant. This indicates that participants still had the image content in their memory.

**Figure 7:**
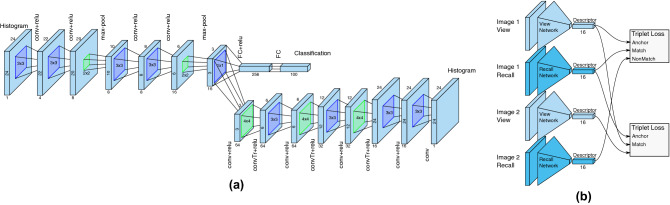
(**a**) CNN architectures employed for direct image retrieval and (**b**) for descriptor learning. Width and height of layers are indicted by the numbers around and below is the number of channels. (**c**) Each histogram was first fed through the network described in (**b**) to generate a 16-dimensional descriptor. Two distinct networks (truncated pyramids) were used for encoding and recall respectively. We used two triplet losses for descriptor learning, forcing matching pairs closer to each other than non-matching ones.

### Data analysis

Each eye movement sequence, either during encoding or during recall, consisted of raw pupil positions on the screen. Collected at a sampling rate of 1000 Hz, we had slightly less than 5000 raw data in each sequence, very often with missing data due to blink. Saccades and fixations were detected from the raw data using the velocity based algorithm provided by SR research (the velocity threshold = $30^\circ /{\text {s}}$ and the acceleration threshold = $8{,}000^\circ /{\text {s}}^2$). Only $95\%$ fixations from eye movements during thinking about an image were located inside of the stimuli domain (SD = $8\%$), while $99\% (SD = 1.9\%)$ fixations during looking at the images were within the stimuli boundaries.

### Data representation

The raw samples from each eye movement sequence were aggregated over time to generate the spacial histogram representation of various sizes. Each grid counts the total number of samples in the cell, which naturally encodes the duration. In total, there were 2800 spatial histograms from perception and 2800 spatial histograms from recall.

### Measures

The performance of a retrieval task can be measured by ranking. In our formulation of a classification task, it means the rank of the true class label. Very often we are not only interested in the top-one performance, but also top-three or top-ten results. Especially in a computational retrieval framework, users’ feedback on the ranking results can be fed back into the methods to improve the overall performance^46,47^. Instead of taking a fixed threshold, we therefore used the full range till top-100 rank as that is the worst rank in a dataset of 100 classes. Connecting the accumulated number of instances in each rank allows to create a Receiver Operating Characteristic (ROC) curve. We took the standard area under the curve (AUC) to measure the accuracy of the classification, i.e. the retrieval accuracy. A curve with a steep slope at the beginning would have a large AUC. The larger the AUC is, the higher the accuracy is. A random guess would correspond to an AUC of $50\%$.

### kNN classification

In order to have a baseline for comparison, we used a simple k-nearest neighbor (kNN) classifier based on Euclidean distance. The basic idea of kNN classifier is each query is exhaustively compared to all training samples and classified as the class of the k-closest ones. k was set to 27 in our experiment and the class label was determined as the distance weighted labels of the nearest 27 samples. The overall accuracy was determined by leave-one-out cross validation. Each time we dropped out one dataset for testing and used all remaining datasets for training. This method was used for image retrieval based on eye movements during encoding and during recall separately.

### CNN classification

For image retrieval from encoding or recall histograms, we employed *Convolutional Neural Networks* (CNN) that operated on histograms of size $24 \times 24$. Each encoding (or, respectively, recall) sequence of eye movements was accumulated into a histogram which served as a one channel input image to the CNN. The classification network was similar to the architecture used in^25^. In the dual-task network (Fig. 7a), a decoder branch that approximately mirrors the structure of the encoder network was added and we skipped the dropout layers as well as batch normalization in both encoder and decoder in the dual-task network. To increase the capacity, a convolution layer was added before each transposed convolution layer in the decoder branch. All weights were initialized using Xavier initialization^48^ and, where applicable, initial filters were orthogonal to each other. The classification network was trained using Cross Entropy Loss (${\mathcal {L}}_{cross}$). The decoder was trained using L1 Loss (${\mathcal {L}}_{L_1}$), which compares the generated histogram to the encoding histogram for the same image. The dual-task network (Fig. 7a) was trained using the combined loss function ${\mathcal {L}} = {\mathcal {L}}_{cross} + \lambda {\mathcal {L}}_{L_1}$, where $\lambda$ is set to 1000, for 80 epochs. We used the Adam parameter update scheme^49^ (learning rate = 5e−4 and weight decay = 2e−5), and a batch size of 100.

To test classification accuracy (in the first two cases), we performed leave one (subject) out cross validation. That is, we trained the network 28 times, each time withholding the data from one of the 28 subjects from the training pool. Testing was then performed only on the data of the withheld subject, thus testing how the network generalizes to new subjects. Overall accuracy was reported as the average over all tests.

### Matching recall sequences to encoding sequences

In order to extend to new images more elegantly, we employed a different formulation to the retrieval problem. Instead of classifying an encoding/recall histogram to an image index, we performed image retrieval by comparing a given recall histogram to a set of encoding histograms of the same subject for which we knew which image was seen. In this setting, image retrieval was cast as a matching problem where the most closely matching encoding histogram indicated which image was recalled.

To this end, we trained an embedding of encoding and recall histograms into a low dimensional descriptor space in which matching encoding and recall pairs were close together, and non-matching pairs were far apart. Figure 7a illustrates a similar network architecture for matching (see more details in^25^) and each (encoding/recall) histogram was mapped to a 16 dimensional descriptor. These descriptors were then used to match a query histogram to a set of previously recorded reference histograms and, by extension, find the closest encoding histogram for a given recall histogram. The difference to the previous network was in the smaller output layer (16 outputs instead of 100) and the removal of the batch normalization layers. Initialization of weights and scaling of the learning rate was handled as in the previous network.

Two triplet losses^27^ were used in tandem with four descriptor mapping networks (Fig. 7b). We trained each fold for 1,000 epochs with an initial base learning rate of 0.001 that we scaled by 0.75 every 100 epochs. We used the same Adam optimizer with $\beta _{1,2} = 0.95$ and a batch size of 20. All layers had a small $L_2$ weight decay of 1e−8. We also experimented with hinge loss, which simply forced matching descriptors to be close and non-matching descriptors apart, but found that triplet loss, while training more slowly, generalized better.

During application, a subject’s encoding sequences for all possible images needed to be recorded and fed through the “encoding-descriptor-network” to compute the descriptors of each image. Then a recall sequence from the same subject could be recorded and fed through the “recall-descriptor-network” to obtain a query descriptor. This query descriptor was then compared to all encoding descriptors to produce a ranking of images based on descriptor distance. At this point, ROC curves and AUC could be computed as in the classification case.

For evaluation, we performed 10-fold cross validation to show that this setup generalizes to new images. The 100 images were split into 10 groups of 10 images each. Training was performed 10 times, each time withholding one group of images from the training data and using it for testing. Reported numbers were the average over the 10-folds.

## Supplementary information

Supplementary file 1

## Data Availability

All data used in the study can be found at project page.

## Code availability

Source code available at project page.
