# Supplementary material for: Computational discrimination between natural images based on gaze during mental imagery
Source: Sci Rep. 2020 Aug 3;10:13035. doi: 10.1038/s41598-020-69807-0 (PMC7400610; doi:10.1038/s41598-020-69807-0)
Supplement: Supplementary file 1 [file 41598_2020_69807_MOESM1_ESM.pdf]

Supplementary Information

Computational discrimination between natural images based on gaze during mental imagery

Xi Wang, Andreas Ley, Sebastian Koch, James Hays, Kenneth Holmqvist, and Marc Alexa

Experiments and Results

Effect of the histogram size. We tested different histogram sizes ranging from 6×6 to 36×36 using kNN. A cell in a 36×36 histogram corresponds to $1.33°$ of visual angle, $1.77°$ in 24×24 histogram and $3.54\circ$ in 12×12 histogram. We performed the two image retrieval tests, namely encoding vs encoding and imagery vs imagery. The achieved accuracy is depicted in Fig. S1 measured by AUC. Among all tested histogram sizes, the best performance of image retrieval based on eye movements during encoding is achieved by using 24×24 histograms with an AUC=95.5%. The performance got better with an increased number of bins from 6 to 24, but got worse with a finer resolution of 36×36. We suspect that the finer differences among individuals are amplified with a finer histogram, and it leads to confusion in such simple Euclidean based k-nearest-neighbor search.

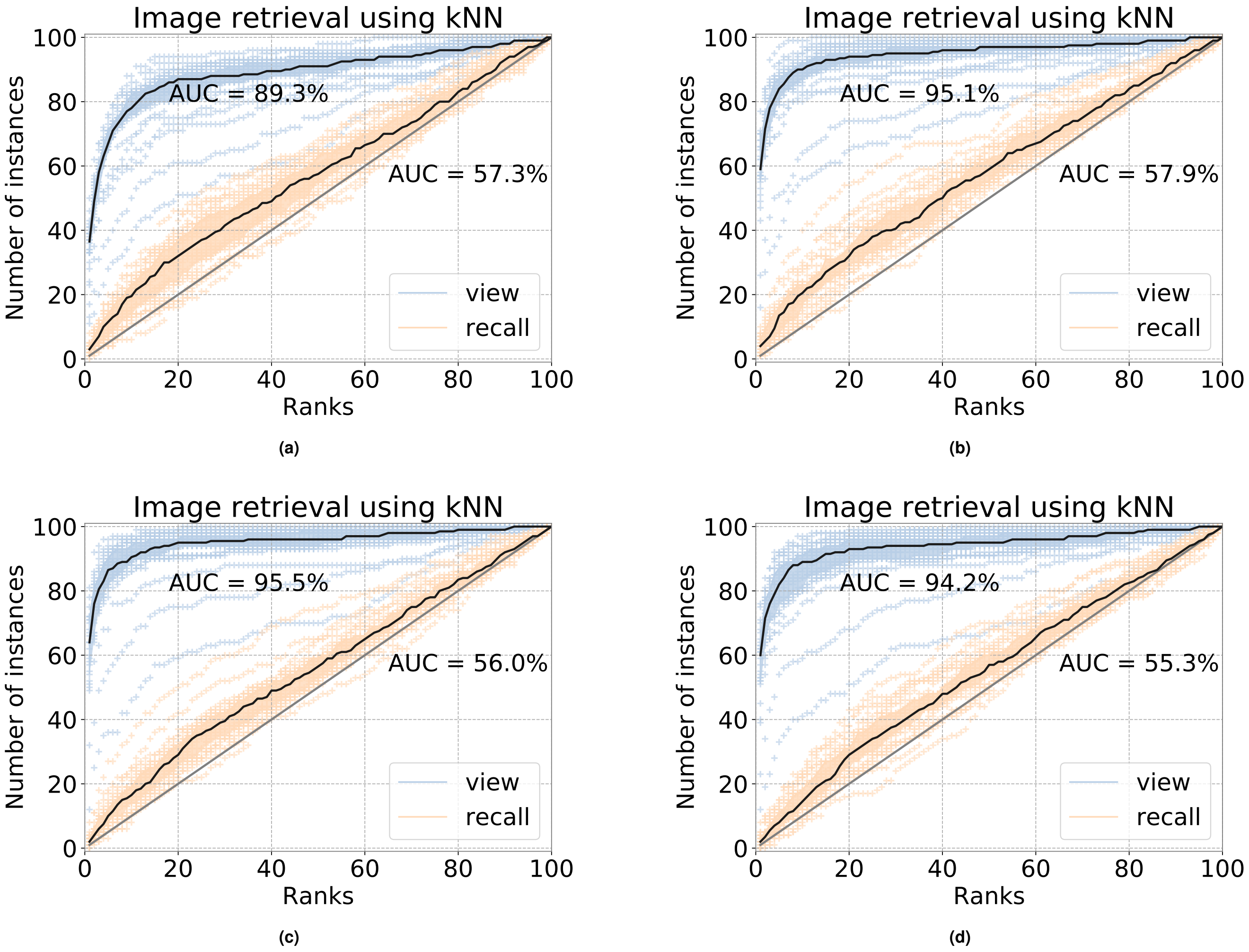

Fig. S1. kNN retrieval accuracy using histograms of 6×6 (a), 12×12 (b), 24×24 (c) and 36×36 (d). Retrieval accuracy of retrieval based on eye movements during encoding are shown in blue and accuracy of retrieval based on eye movements during recall are shown in orange. Black curves are the averaged curve of the leave-one-out cross validation and individual curves are scattered as crosses. Light blue or orange areas mark the center 50% interval.

In contrast, the accuracy of image retrieval based on eye movements during recall was only slightly above chance. The best AUC was 57.9% with histograms of 12×12, which is smaller than the best grid size in the case of encoding vs encoding. Considering the several distortion in eye movements during recall, it is not surprising finer resolution doesn't improve the retrieval accuracy. However, it is reasonable to expect histograms of finer resolution could perform better if we use more

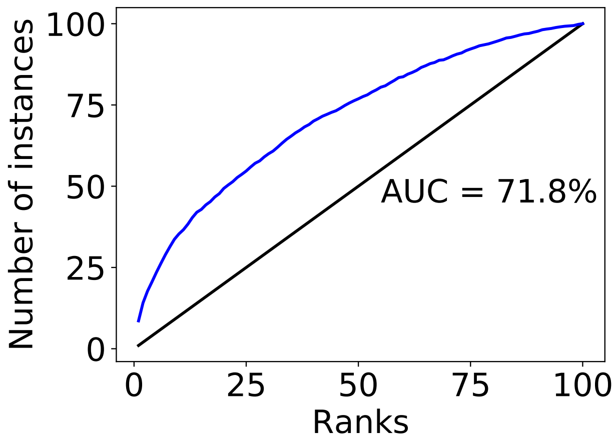

Fig. S2. AUC of image retrieval based on eye movements during recall but using both eye movements during encoding and recall in training. The blue line is the achieved ROC curve with an AUC of 71.8%.

advanced classification methods such as a convolutional neural network. Therefore, in the following analysis, eye movement sequences were represented by histograms of 24×24.

Architecture of the classification network. We initially experimented with a more shallow architecture with less convolutional layers but found that the additional convolutional layers and additional downsampling reduced overfitting, presumably by reducing the number of parameters in the final fully connected layers.

Based on the assumption that eye movements during mental imagery reenact with eye movements during encoding, we tested whether this similarity between gaze patterns during encoding and during recall can improve the performance based on eye movements during recall. Instead of imagery vs imagery, our idea was to include eye movements during encoding in the training data which is encoding + imagery vs imagery. Using the same classification network but more encoding gaze data in training, the achieved AUC was 71.8% as shown in Fig. S2. This suggests that the similarity between gaze data during encoding and during recall can be used in recall based retrieval task and the neural network could potentially benefit from more data.

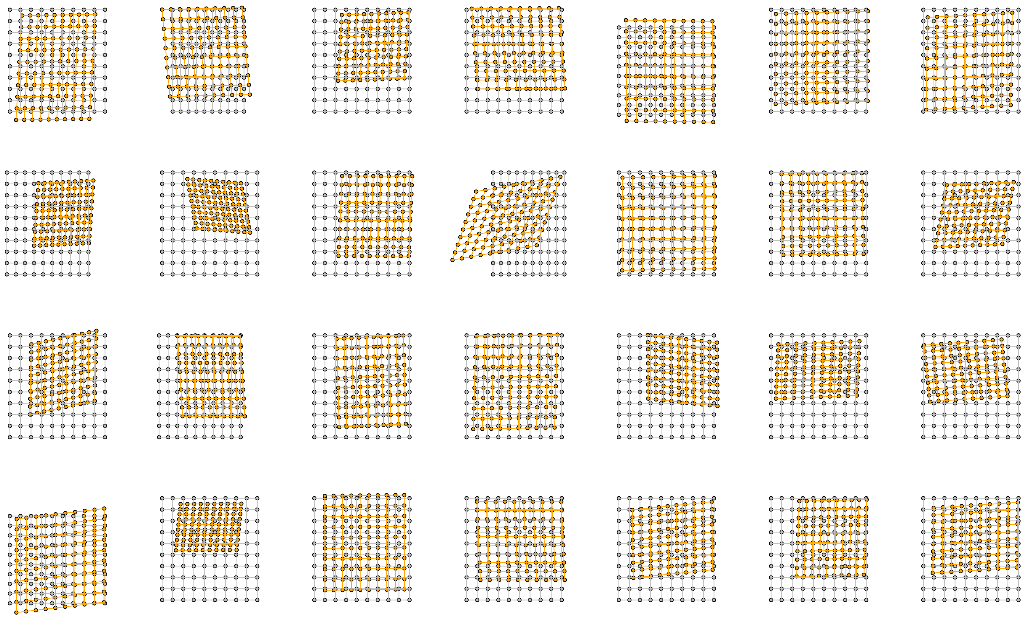

Fig. S3. Distortions between the encoding space and the recall space of each individual data set, visualised by the learned global transformations.

Learning the transformation between encoding and recall. Given a recall gaze histogram, the dual-task network (see Fig. 7a) was designed to predict the image class and to generate the encoding gaze histogram for the same image. The idea was by forcing the decoder network to explicitly learn to map a recall histogram to the encoding one for the same images, the encoder network would learn a better representation that would increase the classification accuracy. However, the dual-task network did not provide a significant boost in performance. In our experiments, adding any dropout layer or using batch normalization would even hurt the testing accuracy of the classification task. We suspect that this has to do with the low signal-to-noise ratio of the data, similar to the effect reported in [1]. Noisy recall data could come from situations when participant did not make extensive eye movements during mental imagery or were distracted by other thoughts in mind. As detailed in [1], training on such data implicitly prevents the network from overfitting.

To understand the non-rigid distortion between the recall space and the encoding space, we computed a global transformation for each dataset of one observer. The transformation was formulated as a moving least squares problem, and the potential local mapping was restricted to be affine. This means we defined the distance to be

$$d_{ij} = ||c_i - Tc'_j||_2^2,$$

where T is an arbitrary affine transformation and d_{ij} represents the Euclidean distance between cell i and j, represented by c_i and c_j. The resulting transformations are shown in Fig. S3, which again illustrates the large variations we have in the data.

More on the triplet loss network. In order to interpret the space of the learned descriptors in the matching network, we visualized all instances of 2800 encoding and recalling eye movement sequences in Fig. S6 using t-SNE (2). Each descriptor is represented by its image in the Figure and ideally images of the same class are close to each other.

Descriptors of encoding sequences of the same image seem to be close to each other as shown in Fig. S6 (a), however, descriptors of recall sequences of the same image are scattered over the space without clear clusters (see Fig. S6 (b)). We believe this to be a side effect of the 10-fold cross validation where in each test fold, only 10 images need to be discriminated from each other. The obtained confusion matrix for each testing fold is visualized in Figure S4. Many of the confusion matrices seem to be biased towards a subset of image instances. The best classification rate was 20 out of 28, and the worst rate was 0. Figure S5 gives the examples of the best retrieval case and one of the worst retrieval case.

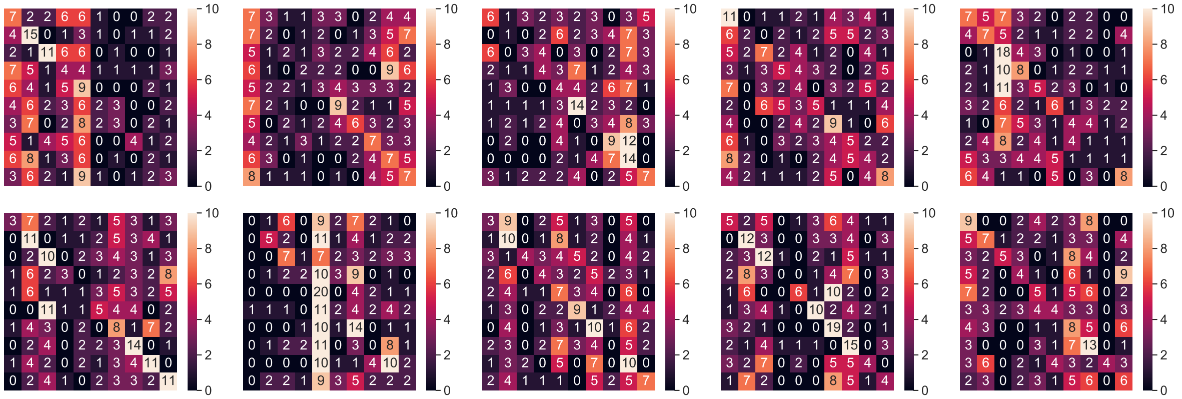

Fig. S4. Confusion matrix for 10-fold cross validation.

The matching paradigm does not quite reach the performance of the classification based approach. A possible explanation is that the histogram pairs contain rather little individual information that could be exploited by a matching approach. The increased opportunity for error, in estimating not only the descriptor for the recall histogram but also for the encoding histogram, could then result in a decrease of performance similar to what we observe.

Data augmentation. By experimenting with data argumentation, we noticed that very slight data augmentation gave a small performance boost, but the performance decreased with an increased strength of argumentation. We suspect that this is related to the variation contained in eye movements during recall. The recall data falls into one of two categories: Recalls with good location precision (fixations close to the gaze fixations) and recalls with very poor location precision. The latter ones were more challenging as our network was trained to largely rely on the location information, and strong augmentation prevents the first type of data from working.

1. Xie L, Wang J, Wei Z, Wang M, Tian Q (2016) Disturblabel: Regularizing cnn on the loss layer in *2016 IEEE Conference on Computer Vision and Pattern Recognition (CVPR).* pp. 4753–4762.
2. Maaten Lvd, Hinton G (2008) Visualizing data using t-sne. *Journal of machine learning research* 9(Nov):2579–2605.
3. Judd T, Ehinger K, Durand F, Torralba A (2009) Learning to predict where humans look in *IEEE International Conference on Computer Vision (ICCV).* pp. 2106–2113.

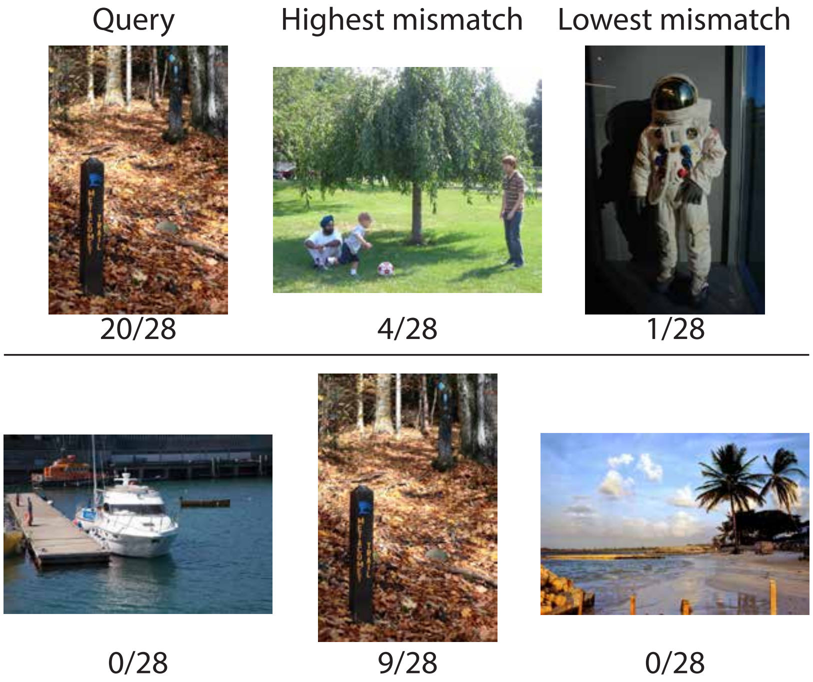

Fig. S5. Mismatching examples using the matching network. Histograms of the query image was matched against the other 10 encoding histograms. The top row shows the examples where the highest matching rate (20 out of 28) was obtained and the bottom row shows one example where the matching rate was 0. The highest mismatching rate was shown in the second column and the lowest mismatching rate was shown in the last column. All images shown here was tested in the same fold. (Public domain imagery downloaded from the dataset published in Judd et al. (3).)

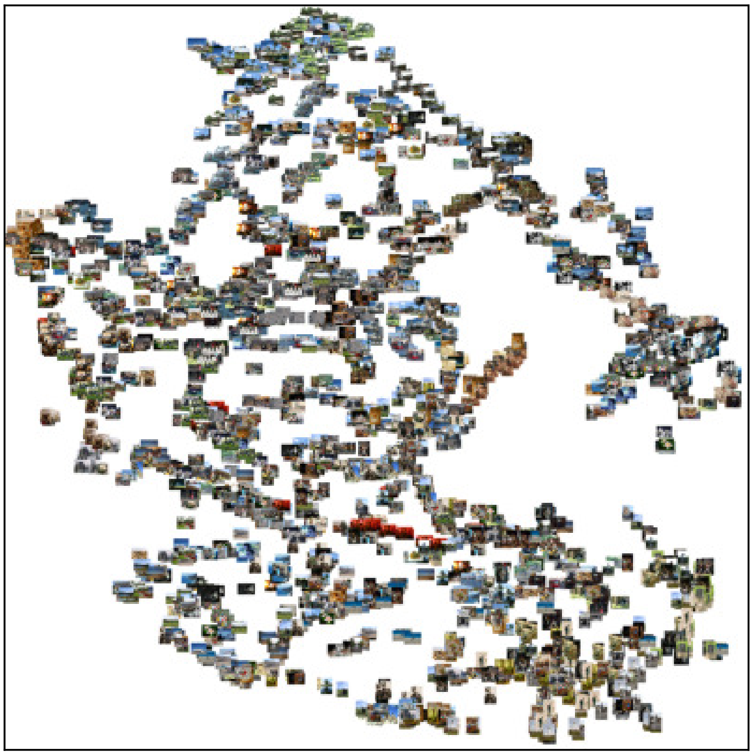

(a)

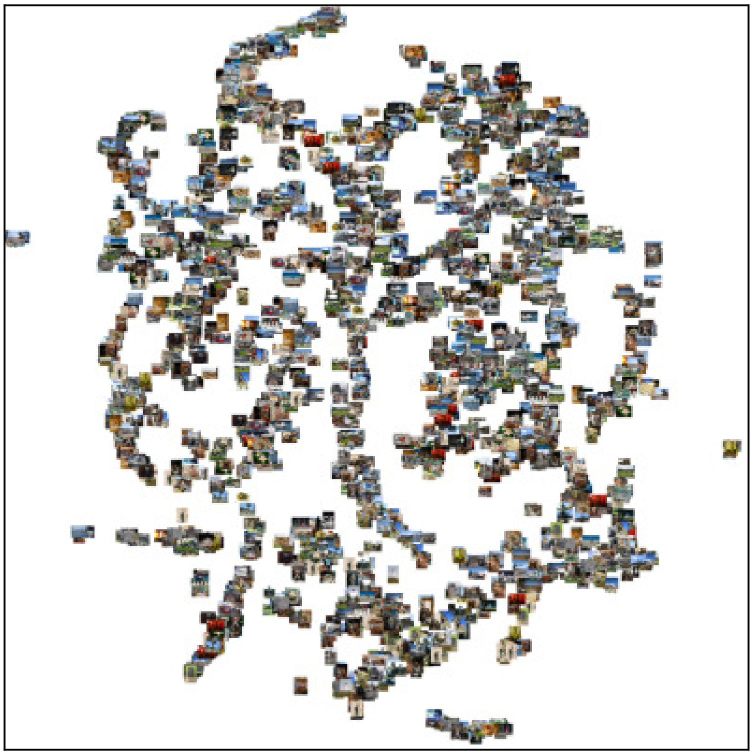

(b)

Fig. S6. Visualization of the descriptors learned by the matching network using t-SNE algorithm. Each descriptor is represented by its corresponding image. The learned descriptors of eye movements during encoding are visualized in (a) and the learned recall descriptors are shown in (b). Ideally descriptors of the same image are close to each other. (Public domain imagery downloaded from the dataset published in Judd et al. (3))
